# Wearable Device-Based Gait Recognition Using Angle Embedded Gait Dynamic Images and a Convolutional Neural Network

**DOI:** 10.3390/s17030478

**Published:** 2017-02-28

**Authors:** Yongjia Zhao, Suiping Zhou

**Affiliations:** 1 School of Automation Science and Electrical Engineering, Beihang University, Beijing 100191, China; 2 School of Science and Technology, Middlesex University, London NW4 4BT, UK; s.zhou@mdx.ac.uk

**Keywords:** gait recognition, gait authentication, gait labeling, angle embedded gait dynamic image, convolutional neural network, biometrics, wearable devices

## Abstract

The widespread installation of inertial sensors in smartphones and other wearable devices provides a valuable opportunity to identify people by analyzing their gait patterns, for either cooperative or non-cooperative circumstances. However, it is still a challenging task to reliably extract discriminative features for gait recognition with noisy and complex data sequences collected from casually worn wearable devices like smartphones. To cope with this problem, we propose a novel image-based gait recognition approach using the Convolutional Neural Network (CNN) without the need to manually extract discriminative features. The CNN’s input image, which is encoded straightforwardly from the inertial sensor data sequences, is called Angle Embedded Gait Dynamic Image (AE-GDI). AE-GDI is a new two-dimensional representation of gait dynamics, which is invariant to rotation and translation. The performance of the proposed approach in gait authentication and gait labeling is evaluated using two datasets: (1) the McGill University dataset, which is collected under realistic conditions; and (2) the Osaka University dataset with the largest number of subjects. Experimental results show that the proposed approach achieves competitive recognition accuracy over existing approaches and provides an effective parametric solution for identification among a large number of subjects by gait patterns.

## 1. Introduction

Gait is cyclical locomotion as a result of human’s natural walk, which reflects a person’s natural or temporary dynamic behavior. Identification or event detection may be achieved based on the differences in gait patterns of different people or that of a same person at different times, wearing different clothes or in different health conditions. Unlike iris, face, fingerprint, palm veins or other biometric identifiers, gait pattern can be collected at a distance unobtrusively [1]. In addition, gait patterns are difficult to duplicate due to their dynamic nature. Over the past decade, gait recognition has attracted increasing attention as a novel biometric technology without the requirement of active cooperation of the subjects. As a result, gait has the potential to be used as a supplement or substitution of existing mainstream biometrics in applications such as social security [2], biometric authentication [3,4,5], health monitoring [6], etc.

Recently, gait recognition using wearable devices has become an active research topic due to the widespread installation of sensors for measuring movement in smartphones [7], smartwatches, fitness bands and other wearable devices. Sensors, which are used for gait data collection, are inertial measurement units mainly consisting of accelerometers and gyroscopes. Most modern accelerometers are tiny triaxial Micro-Electro-Mechanical Sensors (MEMS) that measure acceleration relative to a free fall. Gyroscopes, also known as angular rate sensors or angular velocity sensors, measure the angular velocity in three directions as supplements to acceleration components. With the increasing processing power, nowadays wearable devices with built-in inertial sensors are capable of collecting, processing, and analyzing the gait data in real-time. Hence, gait recognition using wearable devices has become an effective Privacy Enhancing Technology (PET) [8]. By combining the computing and storage capability of the cloud clusters with a huge amount of distributed wearable devices, we can foresee many important applications, especially in the field of identification recognition, which is mainly used for authentication or labeling. Gait authentication can be regarded as a one-class recognition problem by comparing the input gait data with the stored features or built machine learning models of a specific person. Gait labeling is used to identify the subject as the one whose gait patterns in the built models is most like the input gait data. In this paper, we focus on identity recognition by gait patterns using wearable devices, where “wearable devices” refers to smartphones, smart watches, and other wearable devices that are portable and wearable with the capability of measuring user’s movement.

Gait recognition using commercial off-the-shelf (COTS) wearable devices is still immature and full of challenges. In a realistic scenario, the wearable device is always put into the subject’s pocket or worn casually, which means relative orientation between the sensors and the subject’s body cannot be fixed over different sessions of data acquisition. As the coordinate system used by sensors is defined relative to the frame of the device, small orientation changes of sensor installation may make the measurements quite different [9]. Hence, an important issue in gait recognition is modeling the orientation invariant gait features before applying classification methods to the sensor data. Another important issue is robust feature extraction from noisy and complex sensor data which can be easily disturbed by physiological and environmental factors. In this context, feature extraction always needs lots of handcraft work. The third major issue is the selection of classification model. Because the sensor data are collected sequentially with time stamps, classification can be done with some classic methods for time series classification. For example, an effective non-parametric method is to use dynamic time warping (DTW) with k-nearest neighbors (KNN) algorithm. However, such non-parametric methods often need to store all or most training data, which is not applicable when the data size is big. To cope with this problem, machine learning architectures are often used to build elaborate unified models, while a lot of manual preprocessing needs to be done before feeding inertial sensor data to the machine learning architectures.

In this paper, an image-based parametric gait recognition approach using the convolutional neural network (CNN) for training and classification is proposed. The main contributions of this paper are:

- We present a novel 2D representation of gait pattern called Angle Embedded Gait Dynamic Image (AE-GDI), which is inspired by the encoding strategy of Gait Dynamic Image (GDI) illustrated in [9]. There are several advantages of using AE-GDI against the original GDI: (1) It is an orientation and translation invariant dynamic representation for gait pattern; (2). It consists of rich information of temporal distribution and dynamic features from the inertial sensor data time series. By encoding the inertial sensor data time series into AE-GDIs, the problem of time series classification is transformed into an image classification problem, which can then be easily handled by many machine learning algorithms.
- We design an image-based parametric machine learning framework using the convolutional neural network (CNN). As the traditional heuristic methods for feature extraction need lots of human labor and usually rely on experience, it is difficult for them to extract reliable features from AE-GDIs that consist of complex and redundant texture patterns. CNN can extract effective high-level features from input data at the same time of learning classifier parameters during the training phase, so cumbersome hand-craft feature extraction is avoided.
- We propose a grid-based greedy algorithm for gait period detection, which considers both global statistical characteristics and local shape features of the inertial gait time-series signal. The results of gait cycle segmentation are used for aligning and segmenting the AE-GDIs, working robustly in the preprocessing step for gait recognition.
- We evaluate our approach based on two typical gait recognition scenarios, namely, gait authentication and gait labeling, using two public gait datasets respectively.

The paper is organized as follows: in Section 2, we describe the related work in gait recognition. The proposed approach is presented in details in Section 3. In Section 4 we describe our experiment design and present the experimental results. Discussions based the experimental results are presented in Section 5. Conclusions and future work are described in Section 6.

## 2. Related Work

Gait is defined to be the coordinated, cyclic combination of movements that result in human locomotion [10]. The earliest research on gait analysis, which aimed at investigating the normal walking pattern of healthy people, can be traced back to 1960s [11]. Human’s ability to identify people by gait pattern was validated by psychologists in the 1970s. Gait patterns, which were acquired explicitly by recording the pattern of moving lights attached to the body of the subject [12], could be quickly recognized by humans. Since then, a variety of gait acquisition tools and a wide range of research methods have been investigated by researchers in the gait recognition community. And now Gait recognition methods can be classified into three major categories: vision-based [13], floor-sensors-based [14], and wearable-sensors-based [15]. We will focus on inertial sensor-based gait recognition using wearable devices. For an extensive review of gait recognition methods, we refer interested readers to recent survey papers by Sebastijan et al. [16] and Jin et al. [13].

A pioneering research on inertial sensor-based gait recognition was conducted by Ailisto and Mantyjarvi a decade ago [17,18]. In their work, subjects worn a belt with a triaxial accelerometer fixed on it so that the relative position between the sensor and a subject’s body remained unchanged. Only channel x and channel z of acceleration were used because the lateral channel y made little contribution to gait patterns when the subject was walking forward. Then peak detection was used to extract gait cycles, and template matching was employed to identify people. Later, many researches were conducted using sensors fixed in a belt which allows for experimenting with arbitrary sensor installation. However, it cannot be repeated exactly in each experiment even under the same experimental condition.

Nowadays there are many studies where gait data were collected with unconstrained sensor installation which is close to the real scenario. An important issue in these studies is how to ensure orientation invariance when extracting gait features for accurate and robust gait recognition. To this end, an auto-correlation matrix of Fourier transform feature is used to extract orientation invariant gait features in [19]. In [20], a new orientation invariant coordinate system is introduced which aligns with the direction of gravity and motion and then transforms the raw acceleration components into this new coordinate system. Reference [21] estimated the rotation matrix for aligning the probe data by Kabsch optimization. Because data from inertial sensors are described in the local coordinate system, the aforementioned alignment method is not geometrical but statistical. The length and the distribution of the data used to compute rotation matrix or auto-correlation matrix has a significant influence on the results. To address this issue, [9] adopted the inner product or the angle of two three-dimensional measurement vectors of acceleration or angular velocity with a specified time delay as the orientation invariant gait feature and encoded them into Gait Dynamic Image (GDI). However, this gait feature is also vulnerable because it is orientation invariant only when no other transformation is applied on the inertial sensor data. Once the 3D acceleration vectors are translated, which is a necessary step for zero-normalization, the inner product cannot remain unchanged.

Wearable sensor based gait recognition methods can be categorized into two classes: (1) non-parametric methods which are based on the similarity between the probe and stored gallery patterns; (2) machine-learning methods which use a pre-trained model to perform classification tasks given the probe data. In non-parametric methods, different similarity measures have been used, such as Dynamic Time Warping metrics (DTW) [22], Tanimoto distance [21], Euclidean distance [23], etc. Because non-parameter methods require storing all or most gait patterns for all subjects and comparing the probe with all the stored gait patterns, they often lead to low efficiency in computation and storage. However, machine-learning methods can build more compact models than those non-parametric methods. Machine learning algorithms such as hidden Markov model (HMM) classifier [23], Bayesian network classifier [24], support vector machines (SVM), decision trees [24], etc. have been used for a wide range of gait recognition applications. Nevertheless, handcrafted features are widely used in existing gait recognition methods which require extensive experiments to determine the values of some hyperparameters. Unfortunately, the hyperparameters are often problem-specific and sensitive to the specific dataset. In recent years, deep learning has been widely used in the field of human activity recognition [25,26]. In [20], Convolutional Neural Network (CNN) is used to extract features for gait pattern recognition so that labor intensive hand-crafted feature extraction process is avoided.

As can be seen from the previous work, reliable representation of gait pattern and high efficient gait feature extraction are still the major challenges to wearable device based gait recognition. They are also the main motivation for this paper to explore a novel gait representation and design a CNN based parametric classifier for a more effective solution to recognizing people by gait using wearable sensors, which has a wide application prospect in the coming age of ubiquitous computing.

## 3. The Methodology

**Figure 1:**
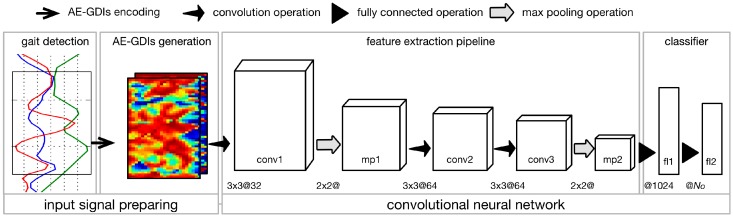
The workflow of the proposed gait recognition approach, which consists of four main modules connected sequentially, namely, gait detection, AE-GDIs generation, feature extraction pipeline, and classifier. The feature extraction pipeline and classifier constitute a complete convolutional neural network. The operations corresponding to different arrows are illustrated above the workflow. Note that “conv”, “mp” and “fl” are the abbreviations of “convolutional layer”, “max pooling layer” and “fully connected layer” respectively. The numbers before “@” refer to the kernel parameters of the corresponding operation and the number after “@” refers to the number of feature maps in convolutional layers, or the output dimension of the fully connected layers.

We introduce an image-based parametric approach for gait recognition using time series signal from inertial sensors mounted in wearable devices, which features angle embedded gait dynamic image (AE-GDI) and convolutional neural network (CNN). The framework of the proposed approach is composed of four basic stages connected sequentially, namely, gait detection (see Section 3.1), AE-GDIs generation (see Section 3.2), feature extraction pipeline and classifier, as shown in Figure 1. The AE-GDI is a novel image-like representation of gait pattern, which is encoded using the linear transformation invariant features of the inertial sensor data series, aiming at reducing the impact of changes of sensor orientation. Because AE-GDIs are segmented using sliding windows with their starting indexes corresponding to the starting positions of the gait cycles, gait detection is a necessary preliminary step for generating AE-GDIs. In the workflow, the last two modules, the feature extraction pipeline and the classifier, constitute a complete CNN (see Section 3.3), which has the ability of automated feature extraction from the input signal without the need of heuristic labor-intensive hand-crafted feature design, and at the same time, performs classification using those extracted features.

We start with the data preparation for the proposed approach. Consider a portable device with built-in inertial sensors (such as a smartphone) that is attached to a person. At time t, the outputs of the accelerometer and gyroscope are denoted by a(t) and q(t) respectively, where a(t), $q\left(t\right)\in {\mathbb{R}}^{3}$. Unlike those stand-alone sensors used under constrained experimental conditions, the sampling frequencies in most wearable devices are time-varying because their processing unit and operating system are designed for multitasking. Therefore, resampling and interpolating steps are needed to transform the raw data into equally spaced time series. The resampling frequency should be large enough to cover the dynamic changes in one complete gait cycle. In this paper, the raw inertial sensor data time series are interpolated using cubic spline and resampled at f = 50 Hz.

For the convenience of further processing, a buffer is used to temporarily hold the incoming sensor data including acceleration time series:

(1) $$a=\{a\left(t\right)|a\left(t\right)={\left[a_{x}\left(t\right)\;a_{y}\left(t\right)\;a_{z}\left(t\right)\right]}^{T},\;t=0:N-1\},$$

angular velocity time series:

(2) $$q=\{q\left(t\right)|q\left(t\right)={\left[q_{x}\left(t\right)\;q_{y}\left(t\right)\;q_{z}\left(t\right)\right]}^{T},\;t=0:N-1\},$$

and acceleration magnitude time series which is generated from time series a:

(3) $$a_{M}=\left\{a_{M}\left(t\right)|a_{M}\left(t\right)=\sqrt{a_{x}^{2}\left(t\right)+a_{y}^{2}\left(t\right)+a_{z}^{2}\left(t\right)},\;t=0:N-1\right\},$$

where $a_{x}\left(t\right),a_{y}\left(t\right),a_{z}\left(t\right),q_{x}\left(t\right),q_{y}\left(t\right),q_{z}\left(t\right)\in {\mathbb{R}}^{1}$. Note that the size N should be carefully determined to ensure the adequacy of gait feature while without the loss of locality. In this paper, N is selected to satisfying l≥4 s empirically (See Section 5 for a discussion of this choice), where l is the time span of the buffer and l=N/f.

### 3.1. Gait Starting Position Detection

A grid-based greedy method for gait starting position detection is presented in this section. The input of the algorithm is the acceleration magnitude data series $a_{M}$. Existing gait cycle detection algorithm is mainly based on a sliding window [21]. However, a sliding window may reject meaningful peaks by simple successive comparison. To tackle this problem, we introduce a constraint to ensure the periodic characteristics of gait cycles using quasi-equally spaced grids that represent the gait cycle starting positions. The grid consists of a series of sequential indexes $h=\left\{h\left(k\right)|k=0:N_{c}-1\right\}$ that partition $a_{M}$ into $N_{c}$ segments which are regarded as gait cycle candidates and h⊂{0:N−1}. The process of gait cycle starting position detection is depicted in Algorithm 1.

Before applying this algorithm, gait period $\hat{g}$ is estimated based on the circular auto-correlation function [21]. We assume that each gait cycle starts with a peak in $a_{M}$. The peaks, which are a set of local maximum points in $a_{M}$, are denoted by a list $p_{M}=\{p_{M}\left(t\right)|t=0:N-1\}$ with the same size as $a_{M}$, where $p_{M}\left(t\right)=1$ if $a_{M}\left(t\right)$ is a local maximum and $p_{M}\left(t\right)=0$ if $a_{M}\left(t\right)$ is not. Each peak is valued using a score that represents the possibility to be the final selected gait cycle starting position. The score is defined as:

(4) $$\tau \left(t\right)=p_{M}\left(t\right)\left(a_{M}\left(t\right)+l^{U}\left(t\right)\right),\;t=0:N-1,$$

where τ(t)=0 if $p_{M}\left(t\right)=0$. For efficiency, τ(t) is computed only when $p_{M}\left(t\right)=1$. $l^{U}\left(t\right)$ is the length of the nearest U-shape to the left of index t. The gait starting position should be aligned with the largest U-shape on its left side in a gait cycle candidate. The length of U-shape is computed by summing up the length of adjacent monotonic decreasing and increasing segments, omitting those with lengths less than a threshold $L_{T}$. The length of a monotonic segment is computed by:

(5) $$L_{j}=\sum_{t=S_{j}}^{E_{j}-1}\sqrt{{\left(a_{M}\left(t+1\right)-a_{M}\left(t\right)\right)}^{2}+\epsilon {f_{s}}^{2}},$$

where $j=1:N_{m}$, $N_{m}$ is the number of monotone intervals in $a_{M}$, and the sign of $\left(a_{M}\left(t+1\right)-a_{M}\left(t\right)\right)$ is the same as $\left(a_{M}\left(S_{j}+1\right)-a_{M}\left(S_{j}\right)\right)$ for $t=S_{j}:E_{j}-1$. $S_{j}$ and $E_{j}$ are the starting index and the ending index for monotone interval j. To treat the time series as a shape described by a series of discrete points, coefficient ϵ is used to adjust the equivalent length of the time interval. Then, $N_{c}$ peaks with high score are chosen to be the gait cycle starting point candidate set C, and $N_{c}$ is the minimum integer that is larger than $2N/\hat{g}$.
**Algorithm 1.** Grid Based Greedy Gait Segmentation Algorithm *1**Input**: ε, C, N// ε is the termination criteria for the iteration2**Initialize**: $c_{0}=\hat{g}$, $b_{0}=0$, i=0, $h_{0}\left(k\right)=b_{0}+\left(k-1\right)c_{0}$ for $k=0:N_{c}-1$3**Do**:// equally spaced grid optimization by iteration4 i=i+1*,*
$N_{c}=\mathrm{int}(N/c_{i-1}$),5**For**
$k=0:N_{c}-1$
**Do**:6  $h_{i-1}\left(k\right)=\mathrm{int}(b_{i-1}+\left(k-1\right)c_{i-1})$,7  $\hat{\varsigma }\left(k\right)$ = *GreedySearch1*($h_{i-1}\left(k\right)$, C),8**End For**9 $c_{i}=\sum_{k=1}^{N_{c}-1}\left(\hat{\varsigma }\left(k\right)-\hat{\varsigma }\left(k-1\right)\right)/(N_{c}-1)$,10 $b_{i}=b_{i-1}+\sum_{k=0}^{N_{c}-1}\left(\hat{\varsigma }\left(k\right)-h_{i-1}\left(k\right)\right)/N_{c}$,11**While**: $\max\left(b_{i}-b_{i-1},c_{i}-c_{i-1}\right)>\varepsilon$12  $N_{c}=\mathrm{int}(N/c_{i}$),13**For**
$k=0:N_{c}-1$
**Do:** //locally optimized without the equally spaced constraint14 $h_{i}\left(k\right)=\mathrm{int}(b_{i}+\left(k-1\right)c_{i})$,15 h(k) = *GreedySearch2*($h_{i}\left(k\right)$, C),16**End For**17**Output**: $h=\{h\left(k\right)|h=0:N_{c}-1\}$* Symbols in Algorithm 1 are described in the context of Section 3.1.

Based on the gait cycle starting point candidate set C, the initial grid defined by its initial starting index $b_{0}$ and spacing $c_{0}$ will be evolved toward them in two main stages until the grid indexes match the corresponding peaks, as shown in Algorithm 1. In the first stage, equally spaced grid optimization by iteration is performed (line 3 to line 11), aiming at estimating the starting index $b_{i}$ and spacing $c_{i}$ of the grid while the grid indexes do not have to correspond to the peaks. In the second stage, each grid index is locally optimized without the equally spaced constraint (line 13 to 16) to move to the nearest peak with higher score. The greedy searching functions in Algorithm 1 are explained as follows:

*GreedySearch 1*: By greedy searching neighborhood of $h_{i-1}\left(k\right)$ within $\hat{g}/2$, return $\hat{\varsigma }\left(k\right)$ which is the nearest starting position candidate with highest score and $\hat{\varsigma }\left(k\right)\in C$,
*GreedySearch 2:* By greedy searching neighborhood of $h_{i}\left(k\right)$ within $\hat{g}/4$, return $\hat{\varsigma }\left(k\right)$ which is the nearest starting position candidate with highest score and $\hat{\varsigma }\left(k\right)\in C$. In this step, the score function is modified by adding a distance penalty:
  (6) $$\tau \left(t\right)=p_{M}\left(t\right)\left(a_{M}\left(t\right)+l^{U}\left(t\right)-\epsilon f_{s}\|t-{\hat{h}}_{t}\left(t\right)\|\right),\;t\in C,$$
  where ${\hat{h}}_{i}\left(t\right)$ is the nearest grid index to t. The distance penalty $\epsilon f_{s}\|t-{\hat{h}}_{i}\left(t\right)\|$ means the score of the starting position candidate decreases with its distance from the nearest grid point.

**Figure 2:**
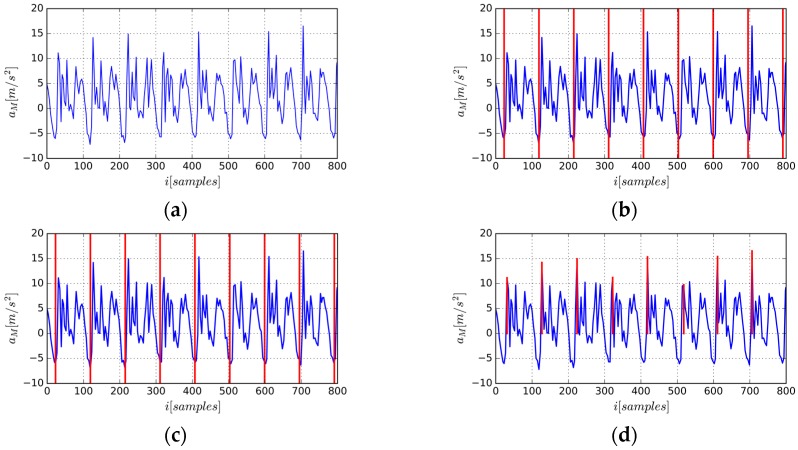
Gait cycle segmentation based on acceleration magnitude time series: (**a**) acceleration magnitude time series; (**b**) equally spaced grid initialization; (**c**) final grid after local searching using greedy strategy; (**d**) corresponding peaks of starting position set of gait cycles, note that there is a “weak” peak in it ensuring gait periodicity.

The process of gait cycle segmentation is shown in Figure 2. Figure 2d is the final segmentation based on the proposed algorithm. Note that the 6th gait cycle starts with a sub-dominate peak, indicating that the grid-based greedy algorithm can provide a robust and accurate gait segmentation because the gait starting position is not only determined by the values of local maxima but also the periodicity constraint defined by the distance penalty item. Unlike [20], resampling the time series to arrive at sequences of fixed lengths is not needed because we only use the starting position of gait cycle as the starting index to generate a new AE-GDI. That is, before generating the representation of gait pattern, time property of raw time series has been kept unchanged, unlike many previous approaches that resampled, filtered, and stretched the resulting gait cycles.

### 3.2. AE-GDI Generation

**Figure 3:**
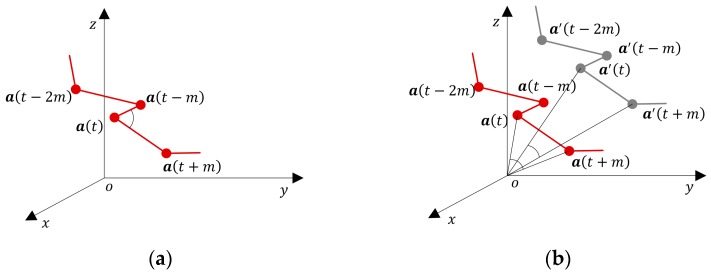
Demonstration for orientation invariant features in 3D subsequence plotted in the sensor coordinate system. (**a**) the angle between vector $\vec{a\left(t\right)a\left(t+m\right)}$ and vector $\vec{a\left(t\right)a\left(t-m\right)}$ used to encoding AE-GDI keeps invariant when rotation, translation and even scaling are applied on a; (**b**) $a^{\prime }$ is a copy of a after applying translation on it. Obviously, the inner product of two acceleration vectors at time t and t+m in $a^{\prime }$ is different from that in a.

AE-GDIs are generated from buffered inertial sensor data series. According to the buffer size and the AE-GDI dimension parameters, a different number of AE-GDIs can be produced. The pixel values of AE-GDI are generated by a function used to determine the meta-features that are invariant to a linear transformation. As linear transformation is angle-preserving, we use the angles formed by the sensor data in the 3D space as the new gait features. We regard the three components of sensor data as the coordinates of a sequence of 3D points in the inertial sensor coordinate system. As shown in Figure 3a, the inertial sensor coordinate system oxyz is a right-handed Cartesian coordinate system in ${\mathbb{R}}^{3}$. Given time delay parameter m, we adopt the angle between vector $\vec{a\left(t\right)a\left(t+m\right)}$ and vector $\vec{a\left(t\right)a\left(t-m\right)}$ as the meta feature, which is defined as:

(7) $$F_{a}\left(m,t\right)=\arccos\left(\frac{\langle a\left(t-m\right)-a\left(t\right),a\left(t+m\right)-a\left(t\right)\rangle }{\left\|a\left(t-m\right)-a\left(t\right)\|\|a\left(t+m\right)-a\left(t\right)\right\|}\right)$$

where t=m:N−m and 0<m<N/2. Geometrically, given any combination of rotation, translation, or scaling transformation to the time series a, $F_{a}$ keep unchanged. In [9], the inner product of two acceleration vectors with time delay parameter m is used to encode Gait Dynamic Image (GDI). Although it is an orientation invariant feature, the inner product is not invariant to translation and scaling which often occur during data normalization and other preprocessing steps, which is illustrated in Figure 3b.

The encoding strategy of AE-GDI is similar to that of GDI [9]. Given row index i and column index j, the pixel value of *k*th one-channel AE-GDI based on acceleration data series a is defined as:

(8) $$\text{AE-GDI}\left(k,i,j\right)=F_{a}\left(m=j,t=h\left(k\right)+i\right)$$

where $i=1:N_{w}$, $j=1:N_{h}$. $N_{w}$ and $N_{h}$ are the time span of AE-GDI and the maximum time delay parameter, respectively. Note that $N_{w}$ and $N_{h}$ also represent the width and the height of the AE-GDI. Following the same procedure, we can get the corresponding one-channel AE-GDIs based on angular velocity q. The number of channels of the final AE-GDIs is denoted by $N_{l}$. Obviously, when only one of a and q is used, $N_{l}$ = 1, while when both a and q are used, $N_{l}$ = 2. To ensure that each AE-GDI contains the gait pattern of at least one complete gait cycle, we align the AE-GDI with the starting position of the corresponding gait cycle so that h(k)∈h and select the time span of AE-GDI $N_{w}$ larger than that of the gait cycle. Generally, a subsequence consisting of k gait cycles can produce k AE-GDIs.

**Figure 4:**
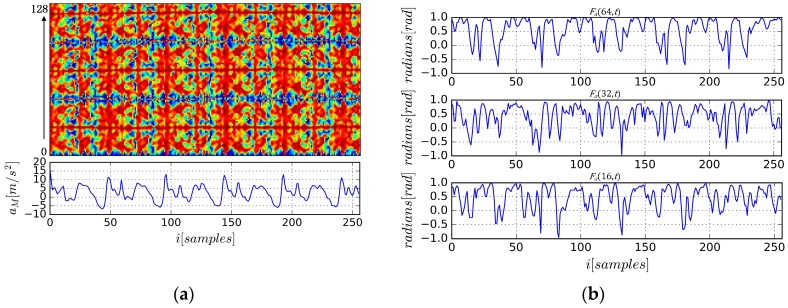
Demonstration for AE-GDI generated from the acceleration components time series, where $N_{w}=256$, $N_{h}=128$; (**a**) AE-GDI and its corresponding acceleration magnitude sequence with the time axis aligned; (**b**) examples for angle time series $F_{a}\left(m,t\right)$ extracted from AE-GDI in (**a**) with m=16,32,64 respectively.

There are some advantages to using AE-GDIs to represent gait patterns. First, as shown in Figure 4a, AE-GDIs reflect the periodicity of the gait data sequence, and they represent much richer 2D features than the original sequences. Figure 4b shows some examples of $F_{a}\left(m,t\right)$ extracted from the AE-GDI in Figure 4a with m=16,32,64 respectively. Thus AE-GDI can be regarded as the integration of several 1D data sequences of the same length tiled side by side. However, the spatial correlation between 1D data sequences contains as much important information as inside the data sequences themselves. This means the AE-GDI shows characteristics more like an image than just the integration of several 1D data. Thus, several image recognition methods can be applied in gait recognition field via AE-GDI representation.

**Figure 5:**
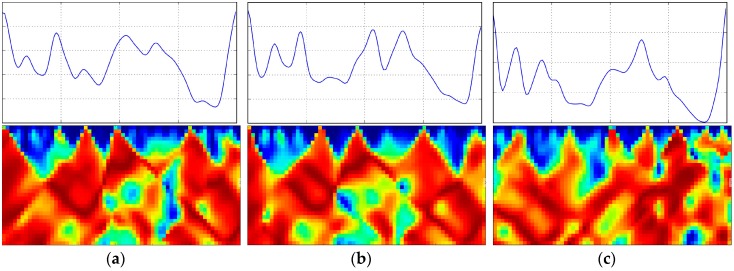
AE-GDIs in the second row and corresponding gait cycles in the first row, where $N_{w}=64$, $N_{h}=32$. (**a**,**b**) are from the same subject and (**c**) is from a different subject.

Second, AE-GDIs are discriminative when representing gait pattern across different subjects. To demonstrate this characteristic, we show three AE-GDIs and corresponding gait cycles in Figure 5. The AE-GDIs in first two columns are from the same subject and that in the third column is from a different subject. We can clearly see the similarity between the AE-GDIs from the same subject and the difference between those from different subjects.

Third, the AE-GDI is a 2D representation of time delay embedding and can be regarded as an effective representation of dynamic characteristics of the non-linear dynamical system based on observation signal. Different from existing work [27,28] which uses the magnitude of acceleration for gait recognition, AE-GDI uses triaxial inertial sensor data and contains rich temporal dynamics, while stays invariant to a linear transformation. In addition, the proposed gait feature is orientation invariant geometrically instead of statistically, leading to robustness to the length of inertial sensor data subsequences.

### 3.3. Convolutional Neural Network

Convolutional Neural Network (CNN) is a type of feed-forward deep neural networks which is typically comprised of one or more convolutional layers followed by several fully connected layers. Different from those ordinary shallow neural networks that are made up of only fully connected layers, CNN itself can act as a feature extractor so that less effort is spent to learn the high-level hierarchy of the input signal by incorporating convolution operation [29]. In this section, we depict the CNN model we designed in details.

#### 3.3.1. Basic Convolution Operation

Convolution is a basic operation in the convolutional neural network. By convolution of the input signal with a linear filter (convolutional kernel), adding a bias term and then applying a non-linear function, a 2D matrix named feature map is obtained, representing local correlation across the input signal. Specifically, for a certain convolutional layer l, the units in it are connected to a local subset of units in the (*l* − 1)th layer. Note that all the units in one feature map share the same weight vector (for convolutional kernel) and bias, hence, the total number of parameters is much less than traditional multilayer neural networks with the same number of hidden layers. This indicates that CNN has a sparse network connectivity, which results in considerably reduced computational complexity compared with the fully connected neural network.

For a richer representation of the input, each convolutional layer can produce multiple feature maps. Though units in adjacent convolutional layers are locally connected, various salient patterns of the input signals at different levels can be obtained by stacking several convolutional layers to form a hierarchy of progressively more abstract features. For the *j*th feature map in the *l*th convolutional layer $C_{l,j}$, the unit at the *m*th row and the *n*th column is denoted as $v_{l,j}^{m,n}$ and the value of $v_{l,j}^{m,n}$ is defined by:

(9) $$v_{l,j}^{m,n}=\sigma \left(b_{l,j}+\sum_{k}\sum_{p_{a}=0}^{P_{l,a}-1}\sum_{p_{b}=0}^{P_{l,b}-1}w_{l,j,k}^{p_{a},p_{b}}v_{l-1,k}^{m+p_{a},n+p_{b}}\right)\;\forall n=1,2,\ldots ,N^{l},\;m=1,2,\ldots ,M^{l}$$

where $M^{l}$ and $N^{l}$ are height and width of feature map $C_{l,j}$. $b_{l,j}$ is the bias of this feature map, k indexes over the set of feature map in the (*l* − 1)th layer, $w_{l,j,k}^{p_{a},p_{b}}$ is the value of convolutional kernel at position $\left(p_{a},p_{b}\right)$, $P_{l,a}$ and $P_{l,b}$ are the size of the convolutional kernel, and σ(⋅) is the Rectified Linear Units (ReLU) nonlinear function. ReLU is defined by:

(10) σ(x)=max(0,x)

Note that different from the CNN often used in computer vision applications, the proposed convolution operation is performed without zero padding. This means each dimension of feature map will be reduced after a convolution operation. Thus:

(11) $$M^{l}=M^{l-1}-P_{l,a}+1\;N^{l}=N^{l-1}-P_{l,b}+1$$

where l is the index of the layer that performs convolutional operation.

#### 3.3.2. Model Implementation

According to the workflow depicted in Figure 1, the proposed CNN is made up of seven sequentially connected layers including three convolution layers (conv1, conv2, conv3), two max pooling layers (mp1, mp2), and two fully connected layers (fl1, fl2) prior to a top-level softmax-group. Max pooling operates independently on every input feature map and resizes it spatially, using the MAX operation. The main advantages of max pooling operation are to progressively reduce the amount of parameters and computation in the neural network, and also to control overfitting. In this paper, the max pooling operations are performed using filters of size 2 × 2 applied with a stride of 2 downsampling every input feature map by 2 along both width and height, leading to 75% of the activations discarded. Another strategy to tackle the overfitting problem is to employ the dropout operation, which is used to set the activation of the hidden neurons to zero with a given probability. In this case, the dropout operation is applied before each fully connected layer during the training procedure and the drop probability is fixed to 0.5.

CNN framework is implemented in tflearn [30] using Tensorflow [31], a high-level library to build and train deep learning networks. For efficiency, the input data are segmented into mini-batches with the size of 100 during training and testing. Thus, an accumulated gradient for model parameters, which are initialized randomly and orthogonally, is derived once for each mini-batch. The parameters of the deep learning framework are optimized iteratively by minimizing the cross-entropy loss function using Adam optimizer and the learning rate is set to 0.001.

During training or testing, AE-GDIs are fed into CNN directly. Note that the number of channels of input AE-GDIs $N_{l}$ = 1 when only accelerometer data or gyroscope data is used and $N_{l}$ = 2 when both the accelerometer and gyroscope data are used. Another undetermined parameter is the dimension of the last fully connected layer $N_{o}$, which will be determined when given a specific gait recognition scenario. In the case of gait authentication, $N_{o}=2$, so that two possible results are produced, acceptance or reject. On the other hand, in the case of gait labeling, $N_{o}$ is the number of subjects. For instance, $N_{o}=744$ when conducting gait labeling on OU-ISIR dataset [32].

As shown in Figure 1, one AE-GDI produces only one possible output. However, in a real scenario, the length of one segment of inertial sensor data series (e.g., the length of the incoming data buffer depicted at the beginning of Section 3) is usually greater than that of one gait cycle. In another word, the label of the subsequence can be co-determined by a series of the AE-GDIs generated from contiguous gait cycles in that subsequence. It is undoubted that the number of AE-GDIs N should be determined carefully because we should consider the case of subject shifting. That is, one sequence of gait data may come from different subjects. To avoid this, N cannot be too large. In the next section, we will evaluate the proposed approach with different values of N.

## 4. Experiment and Evaluation

We conduct experiments on two recently published gait datasets on a PC with an i7-5500U 2.40 GHz CPU and 16 GB RAM. By fed the AE-GDIs generated from the datasets, the CNN model in each run is trained for 50 epochs before we save the model that produces the best results. For each dataset, firstly, we compare the performance with results reported in the literature on the same datasets using other state-of-the-art methods or the designed baseline methods. Then the two gait datasets are used as scenarios for gait authentication and gait labeling respectively, and the corresponding performance is compared using different subsets of the whole datasets.

We adopt the similar performance metrics as used in [26,27]. For gait authentication, we use three performance metrics: precision, recall, and accuracy. Given number of true positive (*TP*), true negative (*TN*), false positive (*FP*), and false negative (*FN*), the precision is defined by:

(12) $$\mathrm{Precision}=\frac{TP}{TP+FP}$$

The recall is defined by:

(13) $$\mathrm{Recall}=\frac{TP}{TP+FN}$$

and accuracy is defined by:

(14) $$\mathrm{Accuracy}=\frac{TN+TP}{TN+TP+FN+FP}$$

For gait labeling, we use labeling accuracy, or the percentage of samples correctly labeled, as the performance metrics.

### 4.1. Performance Evaluation for Gait Authentication on Mobile Phone Dataset

Gait authentication can be regarded as a binary classification problem by comparing the input gait data with the stored features. For the proposed approach, the gait patterns are fed into a parametric classifier prebuilt for the authorized user stored on a local system or authentication server. The quality of the classifier heavily relies on the number of gait patterns used for training the classifiers. To find out the impact of the number of gait patterns in the gallery on the performance of the classifier, the dataset for evaluation should contain enough samples.

#### 4.1.1. The Dataset

The McGill dataset [27] which consists of 40 data files collected from 20 subjects in two days was collected under circumstances that are most close to real-world scenarios [27] among all of the available public gait datasets. The dataset is composed of accelerometer data and gyroscope data from the inertial sensor embedded in the HTC One mobile phone, and the corresponding time stamps from the android operating system. The 10 male subjects and 10 female subjects participated in data acquisition are aged between 19 and 35, with heights between 160 cm and 193 cm. All subjects are asked to perform normal walking with the mobile phone casually put into their left or right pocket. The subjects’ footwear, clothes, and payload may be different between the two-day sessions. Each session lasted for about 15 min. Because of the multitasking nature of the android operating system, the sensor data could not be collected at a fixed sampling rate. The average sampling rate is 28.57 Hz, with a standard deviation of 4.17 Hz [27]. As mentioned in Section 3, before generating gait patterns, the raw sensor data should be resampled into time series with fixed sampling rate. In this case, cubic spline interpolation was used based on the recorded time stamps and the sampling rate is 50 Hz.

During preprocessing, all the gait starting positions are extracted based on single-channel acceleration magnitude $a_{M,id,day}$ (id=1:20, day=1,2) using the proposed grid-based gait detection approach. After that, acceleration vector time series $\left[a_{x,id,day},a_{y,id,day},a_{z,id,day}\right]$ (id=1:20, day=1,2) are encoded into AE-GDIs with window width $N_{w}=64$ and the maximum time delay $N_{h}=32$. So, the resolution of AE-GDI is 32 × 64 and $N_{l}=1$.

#### 4.1.2. Performance Evaluation

To evaluate the proposed approach on this dataset, two situations are considered:

(1) *The same day testing*: training and testing using non-overlapping sensor data from the same day, and for each run of the same day testing, 10% of the whole set in dataset of day 1 are selected randomly as training set while the probe data for testing are selected from the rest of the dataset of day 1 with the same quantity as that of the training data.
(2) *The different day testing*: training and testing using data from different days. For each run of the different day testing, the probe data are the whole set from the data file of day 2 and 10% of the whole dataset from data files of day 1 are used for training.

Similar to [27,33], we evaluate the performance with different number N of consecutive gait cycles as probe data, where N=1,2,3,5. The classifier was built for each subject and evaluated by 10-fold cross-validation. The accuracy of the proposed approach is compared with the previously published approaches in Table 1 where only the results of N=1,2 are shown. To achieve the results in Table 1, [9] used a sliding window of 2.4 s and [33] choose the signal segments of length w = 2.8 s. In this context, our experimental condition was set similar to those in [9,33], because the average length of combined gait cycles when N=2 is 2.14 s.

From the results, we can see that even for N=1 (shorter window length of probe data), the proposed approach outperforms the approach in [9] for the same day testing by 12.2%. If two contiguous gait cycles are used together as the probe data, that is N=2, which makes the probe window length similar to the length of the sliding window in [9] and [33], we can see that the proposed approach outperforms all the baseline methods in term of accuracy. The proposed approach given N=2 outperforms the Cosine Similarity method in [9] by 13.6% for the same day task, and by 5.3% for the different day task. It is obvious that more accurate results can be achieved when the number of consecutive gait cycles N increases.

We can see that the different day task is much more complicated than the same day task because of the changes of clothes, footwear, and payload of the subjects. However, our approach makes a small but deterministic progress on the different day testing. Note that all the feature extraction is accomplished by the CNN model directly, and the parametric model learned is compact. However, both [33] and [9] utilized nearest neighbor classifier to identify each input probe pattern. Nearest neighbor classifier is an effective non-parametric method for recognition and regression while the disadvantage lies in its low efficiency in computation and storage because most of the gallery data need to be stored to compare with the probe data.

#### 4.1.3. Impact of Number of Gait Data Pieces in Gallery

In the McGill dataset, each data file is made up of an approximate 15-min-long inertial sensor time series which is long enough to produce 600–900 valid gait cycles for evaluating the impact of the number of gait data pieces in the gallery on the performance of gait authentication. To do this, we perform the different day testing with the same setting as illustrated above but μ% of the whole dataset from data files of day 1 are selected randomly for training, similar to [33], where μ=10,20,…,100.

**Figure 6:**
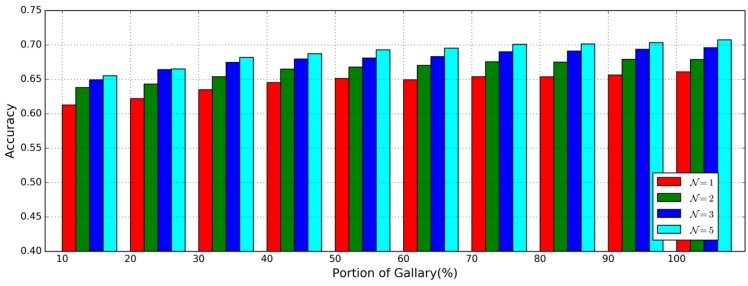
The accuracy of gait authentication on MCGILL dataset given the different proportion of gait patterns in the gallery.

Accuracies with different N and μ in the different day testing are shown in Figure 6 to see the impact of different numbers of gait data pieces in the gallery. As N and μ increase, the accuracies increased slightly. The best accuracy is achieved when N=5 and μ>60 as when μ>60 the performance improvement with μ is very small. This phenomenon is different from that in [33] and tells us that the best accuracy can be obtained using half of the whole training set.

### 4.2. Evaluation for Gait Labeling on the Dataset with the Largest Number of Subjects

Gait labeling is used to identify the subject as the one whose gait patterns in stored features or built models is most like the input. As labeling is a problem of multi-class recognition, we consider the impact of the number of subjects on labeling accuracy in our experiments.

#### 4.2.1. The Dataset

The OU-ISIR dataset [32] is regarded as the largest public inertial-sensor-based walking dataset in biometric recognition community, which is collected using one Motorola ME860 smartphone and three IMUZ sensors for 744 subjects. During the process of data acquisition, the center IMUZ and Motorola were fixed at the center back waist of the subjects. While the other IMUZs were located at the left and right waist respectively. As the IMUZs are dedicated embedded devices for data acquisition, the sampling frequency could be fixed at 50 Hz. As the data are collected under laboratory conditions, the relative position and orientation between the sensors and the subjects’ body are stable. Because this dataset had been divided into the gallery and the probe for all the subjects, we used the given resulting dataset directly.

Although it has the largest number of subjects, the OUIR dataset do not have long periods of inertial sensor data time series for each subject. The average length of gallery sequence is 5.97 ± 1.18 s, while the average length of probe sequence is 4.85 ± 1.10 s. Hence, it is not an ideal dataset for evaluating gait authentication approaches which are mainly based on a parametric machine learning classifier. However, for gait labeling evaluation, this dataset provides adequate subjects. It is interesting to note that we have not found any published gait recognition approach based on a parametric classifier that uses the OU-ISIR dataset to evaluating the impact of the number of subjects on labeling accuracy.

During preprocessing, the AE-GDIs are generated as two-channel images in which the acceleration time series are encoded in channel 1 while the angular velocity time series are encoded in channel 2 with the window width $N_{w}=64$ (corresponding to 1.28 s) and the maximum time delay $N_{h}=32$. Then the resolution of the image pattern is 32 × 64 and $N_{l}=2$.

#### 4.2.2. Performance Evaluation

To assess the performance of the proposed approach for gait labeling, we designed 3 baseline methods by replacing operations in the proposed gait labeling workflow with some existing algorithms. They are described as follows:

- *Method 1*: in the stage of input signal preprocessing, instead of using the Grid based Greedy Gait Detection method (GGGD) proposed in this paper, we apply an Overlap Window strategy [6,34] to segment the raw inertial time-series signal and set the window length to 1.28 s with 0.5 s overlap. Other stages use the same setting as the proposed approach.
- *Method 2*: we substitute the AE-GDI in the proposed CNN framework with GDI which is orientation invariant representation of gait pattern as well [9], while other stages use the same setting as the proposed approach.
- *Method 3*: as the AE-GDIs can be regarded as multi-variate time series, we apply the temporal convolutional kernels [35] on the feature exaction module, by replacing the square kernels (3×3@ for convolutional operation and 2×2@ for max pooling operation) used in the proposed CNN framework in Figure 1 by the 1D kernel along the time axis (1×3@ for convolutional operation and 1×2@ for max pooling operation). We call this modified deep neural work temporal convolutional neural network (TCNN). Other stages use the same setting as the proposed approach.

In the experiments, because the signal segmentation methods are different and the number of samples for each subject are relatively small, we used the whole sequence of each subject as a gallery or probe instead of an independent gait cycle (for GGGD method) or gait data piece (for Overlap Window segmentation), which is the same strategy as used in [32]. The results of gait labeling based on the whole set of the subjects in the dataset are shown in Table 2, and the modified modules are highlighted. In this case, $N_{o}=744$.

From the results, we can see the proposed approach outperformed all the designed baselines, by 56.4%, 18.5%, 8.7% respectively. It is worth noting that the segmentation method is a dominant factor affecting the labeling accuracy. Although we perform a massively overlapped segmentation (0.5 s) attempting to preserve more information than much-less-overlap gait segmentation, the results show segmentation by gait cycles is more effective. By comparing the different representation of gait pattern, we can see that the proposed AE-GDIs provide more accurate features than GDIs, avoiding the turbulence by uncertain experimental condition for each subject. By comparison of the proposed approach and Method 3, AE-GDIs are proved to be more image-like features than time-series-like. Though AE-GDI generation increases the complexity of the proposed approach, it shows opportunities of using lots of available image classification approaches to tackle the gait recognition problems.

#### 4.2.3. Impact of Number of Subjects

**Figure 7:**
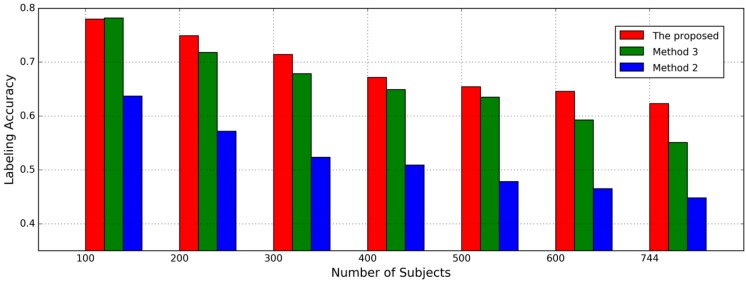
The impact of the number of subjects on gait labeling accuracy. The complete length of inertial sensor data time series in probe dataset is used to determine the labels.

**Figure 8:**
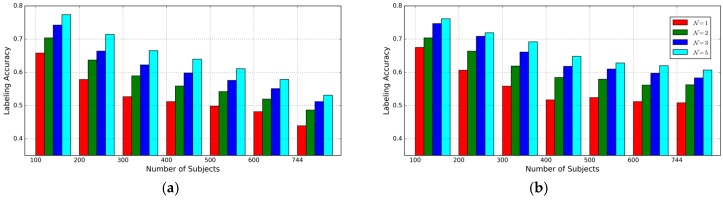
The impact of the number of subjects on gait labeling accuracy given different N values: (**a**) TCNN based Method 3; (**b**) the proposed approach.

The second experiment is designed to evaluate the impact of the number of subjects on labeling accuracy. In this case, the number of subjects K = 100, 200, 300, 400, 500, 600, 744, respectively and complete length of inertial sensor time series in probe dataset is used for labeling. The subsets wth different K are generated by selecting subjects randomly from the whole subject set. As shown in Figure 7, the best accuracy of the proposed approach is 78.0%, while the labeling accuracy decreases as the number of subjects increases. From the results, the proposed approach can achieve more accurate and reliable results than the Method 3 though the latter achieves a better accuracy than the former when the number of subjects is 100. This indicates that the AE-GDI is more like a 2D image than just the integration of 1D data sequence and correlation in time delay angle between different delay may include discriminative information for identifying different subjects. Given different N values, the labeling accuracies based on Method 3 and the proposed approach are shown in Figure 8. We can see that the performance by Method 3 decreases more quickly than that with the proposed approach.

### 4.3. Evaluation for Hyper Parameters of CNN

The CNN used for performance evaluation for gait authentication and gait labeling in Section 4.1 and Section 4.2 is a 7-level deep neural network with the hyperparameters shown in Figure 1. In this section, we evaluate the influence of two key parameters of the CNN framework on performance: the number of convolutional layers $N_{conv}$ and the output dimension of the first fully connected layer $N_{fl1}$.

**Figure 9:**
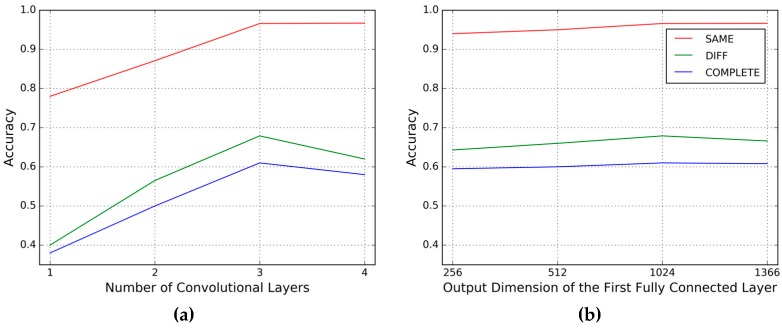
Evaluation for the hyperparameters of the CNN: (**a**) performance on different numbers of convolutional layers; (**b**) performance on different output dimension of the first fully connected layer.

We conduct the same day testing (SAME) and the different day testing (DIFF) on the MCGILL dataset and the complete set testing (COMPLETE) on the OU-ISIR dataset. We fix the first convolutional layer (conv1) and the first max pooling layer (mp1), and watch the influence of the number of the successive convolutional layers before the second max pooling layer, then we have $N_{conv}$ = 1, 2, 3, 4. Note that when $N_{conv}$ = 1, there is no successive convolutional layer following mp1, so the second max pooling layer is then removed. The results are shown in Figure 9.

From the results, we can see that for the same day testing, with the increasing number of convolutional layers and output dimension of the first fully connected layer, the accuracies increase accordingly. While for other testings, we get the best results when 3 convolutional layers are used and the output dimension of the first fully connected layer is 1024, which are the same configuration as shown in Figure 1.

## 5. Discussion

Due to the complexity of human motion, the diversity of factors that affect the gait, and the uncertainty of measurement, there are several unique challenges in the wearable-device-based gait recognition, such as intraclass variability, interclass similarity, etc. All the gait data are similar because they are cyclic multivariate time series, but even for one subject, the gait data may vary significantly with his or her clothes, payloads or health status. For example, the results for one subject in the McGill dataset between the different day testing and the same day testing are very different. Although several attempts have been made to improve the performance of the different day testing, the recognition accuracy is still low.

Nevertheless, as shown in the experimental results, the proposed approach has still made a certain improvement in recognition accuracy and usability, which make it possible to be an effective solution to gait recognition or other problems of one-class or multi-class classification using period time series signal. More specifically: (1) the grid-based greedy method provides a means for a more accurate periodic signal segmentation; (2) the AE-GDI is used to encode rich spatial and temporal characteristics from the raw sensor data into the image-like representation; (3) the use of CNN for automatic feature extraction and classification avoids a lot of labor and empirical knowledge.

It is noticeable that the gait segmentation is essential for a reliable performance, although in human activity recognition (HAR) which is similar to gait recognition, overlap window strategy is more popular for preparing the data. This is because the difference of different classes in gait recognition is much smaller than that in HAR. A high accurate gait segmentation may help the classifier focus on effective gait features instead of their similar periodicity, which is the reason why many researches on gait recognition manage to improve their gait segmenting performance before they extract gait features and design effective classifier.

Our gait segmentation algorithm, which employs periodicity constraint before accurately locating gait starting positions by greedy searching, can effectively avoid misjudgment of fraud gait cycles. Unfortunately, however, it needs a minimum length of the acceleration magnitude time series that is used for gait detection, at least as long as two complete consecutive gait cycles, simply because the grid based periodicity constraint cannot work for the time series including only one gait cycle. At the same time, the length of gait cycles varies with execution time, so the length of the acceleration magnitude time series cannot be too long. In this context, we choose *l* = 4 s.

AE-GDI is a novel 2D representation for gait pattern, which has a similar encoding strategy as GDI but with different meta-feature encodings. The meta features used in AE-GDI are based on the angle between two line segments with the same starting point formed by time delayed sampling on the inertial sensor data sequence. AE-GDI has the form of a two-dimensional matrix, with its rows and columns corresponding to time stamps and time delay values respectively. A comparative study shows our method using AE-GDI as gait representation outperforms that using GDI. In fact, both grid-based greedy gait detection method and AE-GDI encoding should work together to prepare rich discriminative gait signal for the feature extractor in the next stage of the proposed approach.

To extract the high-level features in AE-GDIs efficiently and automatically, CNN is used to build the classifier to identify gait pattern without the need of manually extracting the high-level features. There are many much deeper CNN prototypes available now, for example, GoogLeNet [36] for image classification has 27 neural layers. In early experiments, we found these deep neural networks are prone to cause over-fitting for our experimental scenarios. The over-fitting is mainly caused by the major challenges of gait recognition, i.e., intraclass variability and interclass similarity, so that it is difficult to learn a clear deterministic boundary for gait classification using traditional machine learning methods with hand craft features. In this context, our seven-layer CNN achieves better results with higher computing efficiency. Another finding during designing the CNN framework is that the CNN without zero padding in each convolution operation can obtain slightly better results than that with zero padding.

Computing efficiency is always an important issue in human-involved applications, especially in human-computer interaction scenarios. Considering a case of gait labeling using the complete set of subjects, which is the most complicated case in this paper, the final CNN model has a classifier with 744 classes and consists of 6,268,320 parameters. As a parametric machine learning model, the execution time of CNN has nothing to do with the number of training samples, but only depends on the dimensions of the input feature. We measured the average time of a single run for single sample in the experiment where the resolution of AE-GDI is 32 × 64 and $N_{l}$ = 2, and the execution time is less than 0.02 s, indicating that the proposed method is suitable for real-time applications. Obviously, the performance can be further improved when the proposed approach is implemented and run based on parallel computing platform or other distributed computing environments.

The two public datasets we chose are highly representative: the McGill dataset is close to the real application scenario, while the OU-ISIR dataset has the largest number of subjects. The downside of the OU-ISIR dataset is that it only consists of short-term inertial sensor data. Although we perform training for 50 epochs, the number of the samples is still relatively small. Despite the small sample size, we can still achieve an accuracy of more than 60% for the gait labeling problem with more than 700 classes, indicating that the proposed parameterized gait recognition method has a strong potential for gait labeling application with a large number of subjects.

## 6. Conclusions

In this paper, we proposed an effective parametric gait recognition approach using AE-GDI and CNN. AE-GDI is a novel 2D representation of gait patterns defined based on the linear transformation invariant feature of inertial sensor data sequence. To generate AE-GDIs, a grid-based greedy algorithm is also introduced to achieve robust gait cycle segmentation. The proposed approach is evaluated using the MCGILL dataset with long period inertial sensor data sequence and the OU-ISIR dataset with the largest number of subjects in two main applications of gait recognition respectively: gait authentication and gait labeling. Experiment results show our method is competitive against state of the art approaches or the designed baselines and outperforms them in recognition accuracy. Unlike non-parametric classification, samples in the training set do not have to be stored, and hand-craft selection and extraction of features are not needed with the proposed approach. In addition, the proposed AE-GDI representation allows for some image-based mechanisms like CNN to be applied directly. We believe the proposed approach moves a step toward accurate and reliable wearable-based gait recognition.

Although AE-GDI is orientation and translation invariant for inertial sensor data time series, it is still sensitive to sensor placement. In our future work, we will carry out more experiments for testing and evaluation of our approach on more practical applications to investigate how to improve recognition accuracy based on noisy and complex data from casually and loosely installed sensors. As the convolutional recurrent neural network has proven powerful for both automatic feature exaction and handling temporal correlation, we will investigate how it can be integrated with the AE-GDIs.

## Figures and Tables

**Table 1:** Gait authentication accuracies on the MCGILL dataset.

Approach	Same Day	Different Day
HOS [33]	-	64.5%
Cosine Similarity [9]	85.0%	66.3%
The proposed Approach (N=1)	95.4%	64.9%
The proposed Approach (N=2)	96.6%	67.9%

**Table 2:** Gait Labeling Accuracy in complete subjects set.

Approach	Segmentation	Representation	Deep Neural Network	Accuracy (%)
**Method 1**	**Overlap window**	AE-GDI	CNN	39.0
**Method 2**	GGGD	**GDI** [9]	CNN	42.8
**Method 3**	GGGD	AE-GDI	**TCNN**	56.1
**The Proposed Method**	GGGD	AE-GDI	CNN	61.0
